# Two new species of *Xestoblatta* Hebard, 1916 from Brazil, a redescription of *Xestoblatta roppai* Rocha e Silva Albuquerque & Fraga, 1975 and a key for the species of the *buscki* group (Blattodea, Ectobiidae, Blattellinae)

**DOI:** 10.3897/zookeys.526.6077

**Published:** 2015-10-12

**Authors:** Luiz Rafael Silva-da-Silva, Sonia Maria Lopes

**Affiliations:** 1 Departamento de Entomologia, Museu Nacional, Universidade Federal do Rio de Janeiro-UFRJ, Rio de Janeiro, Brasil

**Keywords:** Key, morphology, new species, taxonomy, *Xestoblatta*

## Abstract

Two new species of *Xestoblatta* from northern Brazil are described, *Xestoblatta buhrnheimi* **sp. n.** and *Xestoblatta rondonensis* **sp. n.**, included in the *buscki* group Gurney (1939), and new characters are added to the description of *Xestoblatta mamorensis* Lopes & Oliveira, 2006. *Xestoblatta roppai* Rocha e Albuquerque-Silva & Fraga, 1975, from midwestern Brazil is redescribed, including its genital characters which were not previously described. Additionally, a key for the species of this group is provided, and photographs are given of the species in the habitus, of tergal modifications, and of the genitalia.

## Introduction

*Xestoblatta* was described by Hebard (1916) with *Xestoblatta carrikeri* designated as the type species. He placed the new genus in the *Ischnopteroides* (with *Symploce* Hebard, 1916, *Ischnoptera* Burmeister, 1838, and *Pseudomops* Serville, 1831). *Xestoblatta* is characterized by morphological characters of the head, the pronotum being ample and without sulci, a widened body, a pale yellow marginal field of the tegmen, and with a neotropical geographical distribution but absent in the West Indies (Hebard 1916). The etymological origin for the generic name *Xestoblatta* is Greek for “polished roach” (Hebard 1916).

Gurney (1939) noted that *Xestoblatta* included a very diverse group of cockroaches and that the 7^th^ abdominal tergites differ in shape among the species. He described eight new species and the males of two species that had been previously described, stressing that male genitalia characters and tergal modifications are important for the generic diagnosis. Additionally, Gurney (1939) described the biology of the species of the genus, documented their geographical distributions, and provided a key to distinguish them. Based on characters such as coloration, number of rami in the ulnar vein, male subgenital plate, paraproct, and male tergal modifications, Gurney split *Xestoblatta* into eight groups based on the shape of the subgenital plate and tergal modifications (Gurney, 1939). One group he described was the *buscki* group, which included *Xestoblatta festae* (Griffini, 1896), *Xestoblatta ramona* Gurney (1939), and *Xestoblatta braziliae* Gurney (1939).

Bruijning (1959) later compiled a key to separate *Xestoblatta*. He distinguished *Xestoblatta* from *Ischnoptera* based on the convex pronotal disk without sulci, tegmina and wings completely developed, and wings widened between discoidal field and anterior surface, with apical triangle wide. He also described a new species (*Xestoblatta surinamensis* Bruijning, 1959) from Suriname.

Rocha e Silva-Albuquerque (1962) described a new species named *Xestoblatta bananae* Rocha e Silva Albuquerque (1962), from Ecuador.

Rocha e Silva-Albuquerque and Fraga (1975) described two new species (*Xestoblatta roppai* and *Xestoblatta vera*) from Brazil.

Grandcolas (1992) noted that species of *Xestoblatta*, found in the litter of neotropical forests, are nocturnal. He also made the observation that the tree hole-dwelling *Xestoblatta cavicola* Grandcolas, 1992 and *Xestoblatta immaculata* Hebard, 1920 are atypical in their gregarious, rather than solitary, behavior. Grandcolas (1992) also characterized *Xestoblatta* by its morphological homogeneity, being monophyletic, and supported the idea that the genital characters and the styles of the subgenital plate (very diversified) form a basis to recognize the various groups within *Xestoblatta*.

Lopes and Oliveira (2007), Pellens and Grandcolas (2008), and Lopes et al. (2012) included *Xestoblatta* in the subfamily Blattellinae based on genital characteristics (i.e. phallomere of the male genitalia being hooked and located on the left side in dorsal view).

Bell et al. (2007) stressed that species of *Xestoblatta*, similarly to other members of Blattaria, are important in the recycling of organic matter.

Lopes and Silva-da-Silva (2014) placed *Xestoblatta iani* Rocha e Silva-Albuquerque, 1964 in *Dendroblatta* Rehn, 1916, in view of the morphology of the pronotum, leg spines, and the configuration of the tergal process of the abdomen and the internal genital plates. These characters supported their placement of the species in Pseudophyllodromiinae based on the position of the hooked structure of the male genitalia.

Evangelista et al. (2015) listed the species of cockroaches known from the Guiana Shield, based on literature records and field collection, which included a new species, *Xestoblatta berenbaumae*.

*Xestoblatta* includes 43 species, all of which are neotropical (Lopes et al. 2012; Beccaloni 2015; Evangelista et al. 2015). They occur in the United States, south to Bolivia and southern Brazil. In Brazil the genus is represented by 17 species.

In this paper we provide additional characters to define the *buscki* group (Gurney, 1939), provide a key for the species of this group, and describe two new species from the states of Amazonas and Rondônia. Two previously described species are also newly included in the group and are redescribed, including new information on their genital structures, (*Xestoblatta roppai* Rocha e Silva Albuquerque & Fraga, 1975) and paraprocts (*Xestoblatta mamorensis* Lopes & Oliveira, 2006).

## Material and methods

The genital plates were removed after dissection of the posterior part of the abdomen, using traditional dissection techniques, for examination (Lopes and Oliveira 2000) (*Xestoblatta roppai* and *Xestoblatta mamorensis* had been previously dissected and the genitalia were mounted on a slide). After study, the genitalia of all species were stored in glycerin in micro-vials and attached to the respective sample, follow Gurney et al. (1964). The terminology for the genitalia and the taxonomic classification follows Roth (2003). The specimens were compared with other specimens of *Xestoblatta* deposited in the Blattaria Collection of the Museu Nacional of the Federal University of Rio de Janeiro (MNRJ). The types of *Xestoblatta roppai* and *Xestoblatta mamorensis* deposited in the Blattaria collection of the Museu Nacional/UFRJ, were examined and compared with descriptions in the literature. Digital images of the habitus, pronotum, head and genitalia were taken with a camera mounted on a stereoscopic microscope. The descriptive terminology follows Beier (1970). The holotypes of *Xestoblatta buhrnheimi* sp. n. and *Xestoblatta rondonensis* sp. n. are deposited in the collection of the Department of Entomology at the Museu Nacional of Rio de Janeiro (MNRJ).

## Results

### Blattodea Brunner, 1865
Ectobiidae Brunner von Wattenwyl, 1865
Blattellinae Karny, 1908
*Xestoblatta* Hebard, 1916 *buscki* group

**Diagnosis.** The males of the species in this group have the subgenital plate trapezoidal in shape, regular or irregular, with accessory styles on both sides of the plate; tergal modification in the 7^th^ tergite; left paraprocts on the supra-anal plate developed and hooked, reaching beyond half the length of supra-anal plate, with or without setae.

Species included: *Xestoblatta buhrnheimi* sp. n.; *Xestoblatta rondonensis* sp. n.; *Xestoblatta mamorensis* Lopes & Oliveira, 2006; *Xestoblatta roppai* Rocha e Silva & Fraga, 1975 (transferred from *castanea* group to *buscki* group in this paper).

**Remarks.** Rocha e Silva Albuquerque and Fraga (1975) placed *Xestoblatta roppai* in the *castanea* group and considered its general aspect similar to *Xestoblatta para* Hebard, 1926 and *Xestoblatta nyctiboroides* (Rehn, 1906). Based on analysis of the holotype and its genitalia, which had not been described in the original description, we have concluded that it should be included in the *buscki* group, in view of the morphological similarities such as in the subgenital plate, styles, and left phallomere.

**Key for the identification of males of species in the *buscki* group of *Xestoblatta***

**Table:** 

1	Paraproct bifid, without setae or with only a few sclerotized setae; one apex slender, the other convex	**2**
–	Paraproct bifid or not, with sclerotized setae resembling spines	**3**
2	Left style slender, simple, with bifid accessory style; Body mostly brown	***Xestoblatta mamorensis* Lopes & Oliveira, 2006**
–	Left style slender, bifid, with accessory style simple; Body mostly dark brown	***Xestoblatta rondonensis* sp. n.**
3	Right style trapezoidal, with below 6 spiniform projections	**4**
–	Right style trapezoidal, with 6 spiny projections	***Xestoblatta roppai* Rocha e Silva Albuquerque & Fraga, 1975**
4	Right style with two small lateral projections, with spiny apex	***Xestoblatta buscki* Gurney, 1939**
–	Right style with three larger projections, two next to one another. All with spiny apex	***Xestoblatta buhrnheimi* sp. n.**

#### Xestoblatta buhrnheimi sp. n.

Taxon classificationAnimaliaBlattodeaEctobiidae

http://zoobank.org/C3B1EE44-7AE8-4605-AEE3-7119CD3739DE

Figs 1–11

##### Type material.

Holotype ♂. BRAZIL, Amazonas, Coari, Rio Urucu, ROC 27’ - 4°49'34"S/ 065°15'37"W, 05–18/03/1994. P.F. Bührnheim et. cols. (Shannon trap), in MNRJ.

##### Diagnosis.

This species is characterized by having supra-anal plate with lower margin with two small medio-lateral protuberances; left paraproct slender, weakly sclerotized, folded on itself; right paraproct hooked, strongly sclerotized, round apically, reaching beyond half of plate and covered with spines ventrally; genitalia with left phallomere hooked, concave, sclerotized, slender apically;. median sclerite long, slender, with lanceolate tip (Fig. 10). Right phallomere triangular medially, weakly sclerotized and bearing convex sclerotized structure apically.

**Figures 1–11.**
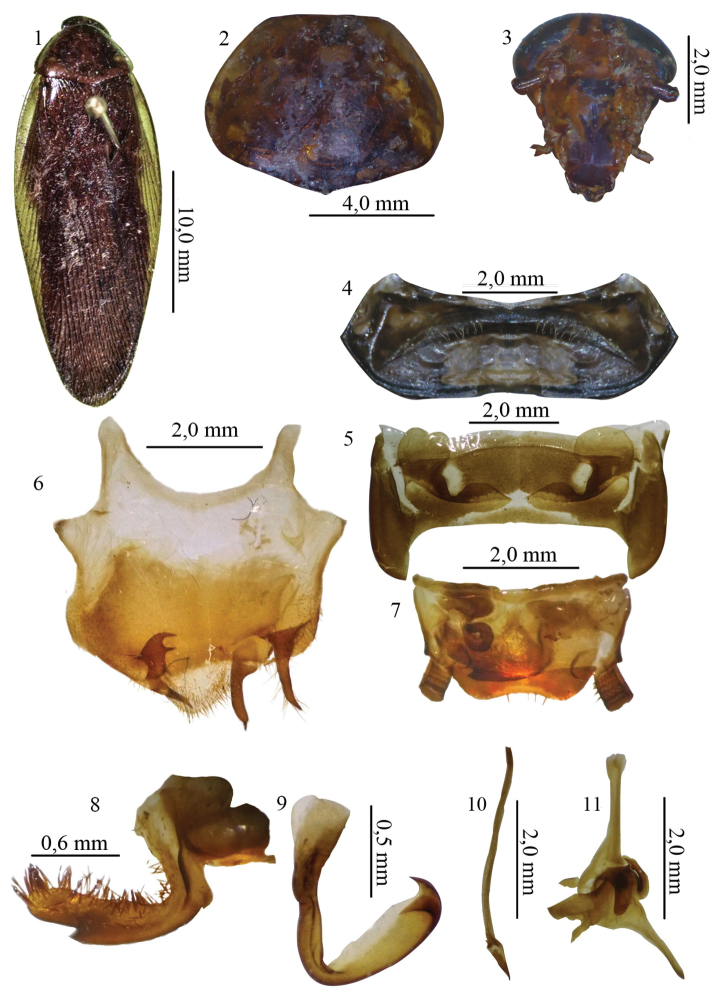
**1** Habitus, dorsal view, of the species *Xestoblatta buhrnheimi* sp. n. holotype male (MNRJ) **2** Pronotum, dorsal view, holotype male (MNRJ) **3** Head, ventral view, holotype male (MNRJ) **4** Tergal modification of the tergite I, dorsal view, holotype male (MNRJ) **5** Tergal modification of the tergite VII, dorsal view, holotype male (MNRJ) **6** Subgenital plate, ventral view, holotype male (MNRJ) **7** Supra anal plate, dorsal view, holotype male (MNRJ) **8** left paraproct, dorsal view, holotype male (MNRJ) **9** left phallomere, dorsal view, holotype male (MNRJ) **10** median sclerite, dorsal view, holotype male (MNRJ) **11** right phallomere, dorsal view, holotype male (MNRJ).

##### Description.

Holotype. Male dimensions (mm): total length: 28. length of pronotum: 5.3; width of pronotum 7.4; length of tegmen: 24.1; width of tegmen 6.1. General coloration brown (Fig. 1). Pronotum light brown, shiny, with small irregular marks (Fig. 2). Head with vertex yellowish; palp light brown; antenna with apical segments dark brown (Fig. 3). Legs yellowish brown with dark brown spines. Pulvilli milky yellow. Arolia brown. Tegmen with anal field dark brown, lateral flap yellowish brown. Abdomen dark brown with white marks on medial area of first tergites. Supra-anal plate and subgenital plate yellowish brown.

Head. Triangular; interocular space narrow, occupying more than half the space between antennal insertions; vertex completely exposed. Ocelli small and little differentiated. Antennae long, slender, reaching beyond tip of abdomen; last segment of maxillary palp setose, 5^th ^segment subequal to 4^th^ segment.

Thorax. Pronotum convex and subtrapezoidal, apex straight, base slightly angular with lateral flaps slightly deflexed and margins round, widest at mid-caudal region. Disk of pronotum without sulci. Fore femur on anteroventral surface with 12 spines decreasing in size and two apical spines; mid femur on anteroventral surface with five spines decreasing in size and three spines increasing in distally; geniculate spine present; posteroventral surface with four subequal spines. Hind femur on anteroventral surface with seven subequal spines, plus one apical spine and one geniculated spine. Mid and hind coxae with latero-apical projections on inner surface. Arolia present. Claws symmetrical and without specialization. Tegmen developed, reaching beyond apex of abdomen, wider in anterior 1/3; marginal field well marked and slightly concave; scapular field slightly widened and round; discoidal field ample, anal field wide, with 8 axillary veins. Wings developed; ulnar vein with five incomplete rami and seven complete rami; apical triangle not developed; anal field folded as a fan.

Abdomen. Tergite I bearing median concavity with a series of marginal setae (Fig. 4). Tergite VII with two sulci on lateral margins, which are hidden by expansion of tergite VI (Fig. 5). Subgenital plate widened, with lower border setose, apex “V”shaped; left style small, wide, sclerotized with 2-3 apical spines; right accessory style similar to right style, and inserted next to it; left style small, wide, sclerotized, with 2-3 apical spines; left accessory style slender, shorter than other styles (Fig. 6). Supra-anal plate with lower margin with two small medio-lateral protuberances (Fig. 7). Left paraproct slender, weakly sclerotized, folded on itself; right paraproct hooked, strongly sclerotized, round apically, reaching beyond half of plate and covered with spines ventrally (Fig. 8). Genitalia with left phallomere hooked, concave, sclerotized, slender apically (Fig. 9). Median sclerite long, slender, with lanceolate tip (Fig. 10). Right phallomere triangular medially, weakly sclerotized and bearing convex sclerotized structure apically (Fig. 11).

##### Remarks.

This species is close to *Xestoblatta roppai* in size, in the paraproct covered with spines ventrally, and modifications of tergite VII. It differs in the shape of the subgenital plate and the paraproct is longer and more slender than in *Xestoblatta roppai* (Figs 39 and 41).

##### Etymology.

The species was named in honor of Frederico Bührnheim, collectors of the specimens.

##### Known geographical distribution.

Brazil (AM)

#### Xestoblatta rondonensis sp. n.

Taxon classificationAnimaliaBlattodeaEctobiidae

http://zoobank.org/B724266D-065E-4926-801B-3F073D1BD5CF

Figs 12–22

##### Type material.

Holotype ♂, Brazil, Rondônia, Parque Estadual Guajará-Mirim, 26/01/1998, M.C. Araújo, Robson, Laurivite & João Raimundo leg. Atrás do acampamento. Paratypes: 1 ♂ and 2 ♀, same data as the holotype, and 3 ♀, Reserva Mamoré, trilha atrás do acampamento (trail behind campsite) MNRJ.

##### Diagnosis.

This species is characterized by having subgenital plate with margin ciliated, styles inserted laterally and spiny projection below left style. Right style rectangular with seven small sclerotized spines, inserted on lateral surface of style; left style short, not extending to 1/5 of subgenital plate, bifid, claw-shaped; accessory style present, curved, pointed, smaller than right style. Supra-anal plate trapezoidal with margin setose, cerci with 19 segments. Right paraproct long, extended beyond half of supra-anal plate, with two apical lobes, one slender and the other convex. Genitalia with left phallomere hook-shaped and with apical projection sclerotized; median sclerite slender, pointed, with slight apical curvature; right phallomere shaped as an inverted “Y” with sclerotized base.

##### Description.

Holotype. Male dimensions (mm) holotype ♂: Total length: 26.0; length of pronotum: 5.4; width of pronotum: 6.8; length of tegmen: 23.5; width of tegmen: 6.5.

**Figures 12–22.**
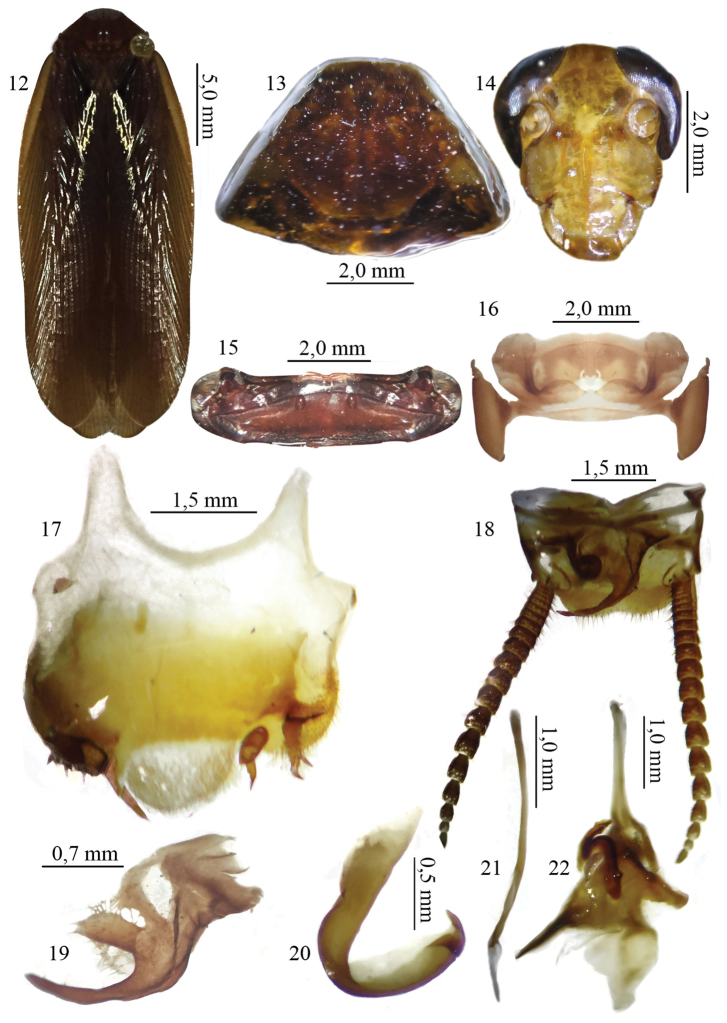
**12** Habitus, dorsal view, of the species *Xestoblatta rondonensis* sp. n. holotype male (MNRJ) **13** Pronotum, dorsal view, holotype male (MNRJ) **14** Head, ventral view, holotype male (MNRJ) **15** Tergal modification of the tergite I, dorsal view, holotype male (MNRJ) **16** Tergal modification of the tergite VII, dorsal view, holotype male (MNRJ) **17** Subgenital plate, ventral view, holotype male (MNRJ) **18** Supra anal plate, dorsal view, holotype male (MNRJ) **19** right paraproct, dorsal view, holotype male (MNRJ) **20** left phallomere, dorsal view, holotype male (MNRJ) **21** median sclerite, dorsal view, holotype male (MNRJ) **22** right phallomere, dorsal view, holotype male (MNRJ).

General coloration. Dark brown and shiny (Fig. 12). Pronotum with lateral flaps light brown, contrasting with disk (Fig. 13). Head light brown (Fig. 14); antennae and maxillary palp golden tomentose. Tegmen with marginal field light brown. Legs reddish-brown and shiny; pulvilli whitish.

Head. Triangular and small; vertex covered by pronotum in dorsal view, interocular space ample, about 2/3 distance that separates antennal insertions; ocelli large and conspicuous; antennae long and ciliated, extending past apex of abdomen; maxillary palp ciliated, 3^rd^ segment longer than the others, 4^th^ segment slightly smaller than 5^th^, both dilated, 4^th^ segment expanded apically and 5^th^ basally.

Thorax. Pronotum convex and subtrapezoidal, with apex straight, base slightly angular and lateral flaps deflected, with round borders. Disk of pronotum without sulci. Tegmen long, extending beyond apex of abdomen; marginal field narrow and elongated, scapular field long with veins obliquely arranged; discoidal field ample, with veins arranged longitudinally and anal field elongate, with 8–9 axillary veins. Wings developed; apex of radial vein rami and costal field not dilated; anal field fan-folded and with small apical triangle. Legs long and spiny; fore femur on anteroventral surface with four spines up to median region, followed by series of strong spines that gradually decrease in size toward apex, and three large apical spines; posteroventral surface with five developed spines, one apical. Mid and hind femora with ventral surfaces similar, with seven developed spines, spaced, one apical; genicular spine present; pulvilli present on all tarsal segments; arolia developed; claws symmetrical and not specialized.

Abdomen. Tergite I modified, bearing row of setae (Fig. 15) and segment VII with medio-lateral concavity (Fig. 16). Subgenital plate with margin ciliated, styles inserted laterally and spiny projection below left style. Right style rectangular with seven small sclerotized spines, inserted on lateral surface of style; left style short, not extending to 1/5 of subgenital plate, bifid, claw-shaped; accessory style present, curved, pointed, smaller than right style (Fig. 17). Supra-anal plate trapezoidal with margin setose, cerci with 19 segments (Fig. 18). Right paraproct long, extended beyond half of supra-anal plate, with two apical lobes, one slender and the other convex (Fig. 19). Genitalia with left phallomere hook-shaped and with apical projection sclerotized (Fig. 20); median sclerite slender, pointed, with slight apical curvature (Fig. 21); right phallomere shaped as an inverted “Y” with sclerotized base (Fig. 22).

##### Remarks.

This species is similar to *Xestoblatta mamorensis* in the bilobed right paraproct with one slender lobe, and the distribution of styles on the subgenital plate. It differs in the shape of the right style (Fig. 17), coloration and habitus (Fig. 12), and right phallomere (Fig. 22)

##### Etymology.

The species name honors the state of Brazil where the species was collected.

##### Known geographical distribution.

Brazil (RO)

#### Xestoblatta mamorensis

Taxon classificationAnimaliaBlattodeaEctobiidae

Lopes & Oliveira, 2006

Figs 23–33

##### Type material.

Holotype ♂ (examined) Brasil: Rondônia, Reserva Mamoré, 25/01/1998, without collector. Museu Nacional, UFRJ, Rio de Janeiro, Brazil.

##### Dimensions

(mm). Holotype ♂ Total length: 30; length of pronotum: 5.0; width of pronotum: 8.0; length of tegmen: 25; width of tegmen: 7.0.

##### Description.

**Figures 23–33.**
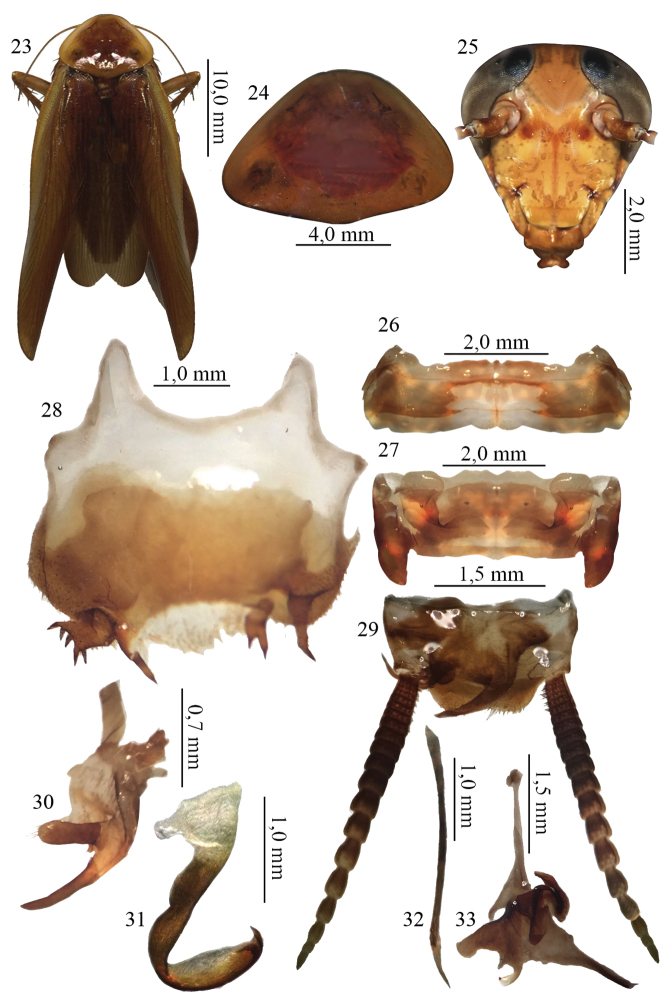
**23** Habitus, dorsal view, of the species *Xestoblatta mamorensis* Lopes & Oliveira, 2006. holotype male (MNRJ) **24** Pronotum, dorsal view, holotype male (MNRJ) **25** Head, ventral view, holotype male (MNRJ) **26** Tergal modification of the tergite VII, dorsal view, holotype male (MNRJ) **27** Tergal modification of the tergite VII, dorsal view, holotype male (MNRJ) **28** Subgenital plate, ventral view, holotype male (MNRJ) **29** Supra anal plate, dorsal view, holotype male (MNRJ) **30** right paraproct, dorsal view (MNRJ) **31** left phallomere, dorsal view (MNRJ) **32** median sclerite, dorsal view MNRJ **33** right phallomere, dorsal view, of the holotype (MNRJ).

This species was found in the Reserva Mamoré, Rondônia. It can be separated from other *Xestoblatta* species by the habitus (Fig. 23), coloration of the pronotum (Fig. 24) and head (Fig. 25), shape of the tergal modifications (Figs 26 and 27), and morphological differences in the subgenital plate (Fig. 28). In the original description, tergite VII and the right paraproct were not described. These characters are deemed very important to separate the species and therefore are described below.

Abdomen. Tergite VII with two lateral grooves (Figs 26 and 27). Supra-anal plate with right paraproct reaching beyond half of plate (Fig. 29). Also with two lobes, one convex at apex and covered with weakly sclerotized setae, and the other slender, curved and larger than the first (Fig. 30). Genital plate with left phallomere hooked (Fig. 31); median sclerite slender, lanceolate (Fig. 32); right phallomere shaped as inverted “Y” and with sclerotized base (Fig. 33).

##### Known geographical distribution.

Brazil (RO)

#### Xestoblatta roppai

Taxon classificationAnimaliaBlattodeaEctobiidae

Rocha e Silva & Fraga, 1975

Figs 34–44

##### Type material.

Holotype ♂ – Brazil: Mato Grosso, Vila Vera, X-1973, Roppa & Alvarenga col. Museu Nacional, UFRJ, Rio de Janeiro, Brazil.

##### Dimensions

(mm). Holotype ♂ Total length: 26 to 30; length of pronotum: 4.0; width of pronotum: 5.9; length of tegmen: 22.3; width of tegmen: 11.4.

##### Description.

**Figures 34–44.**
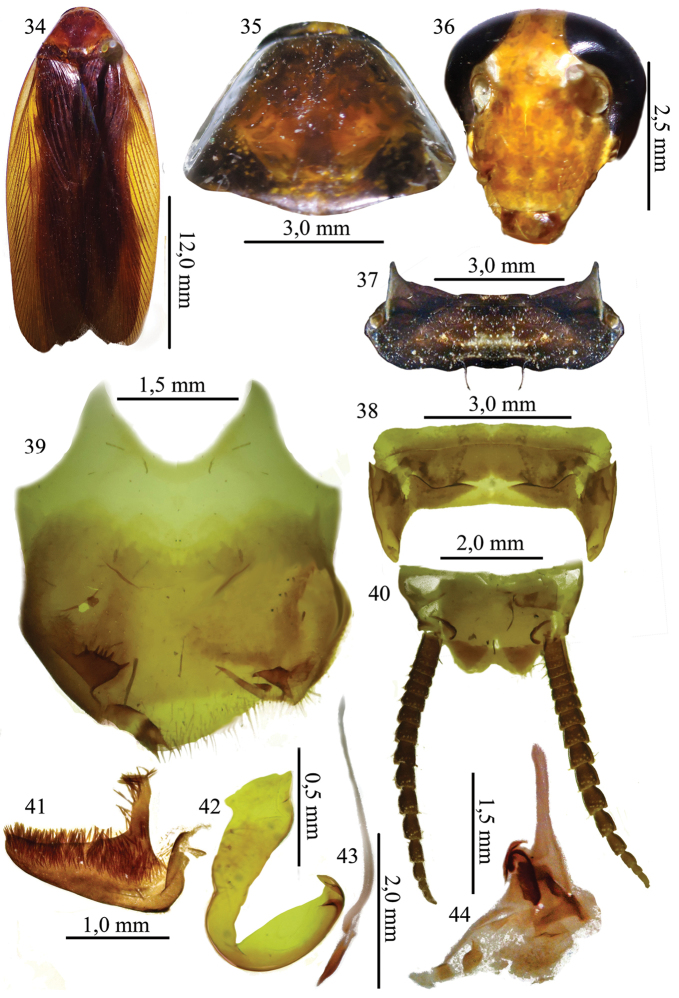
**34** Habitus, dorsal view, of the species *Xestoblatta roppai* Rocha e Silva & Fraga, 1975. holotype male (MNRJ) **35** Pronotum, dorsal view, holotype male (MNRJ) **36** Head, ventral view, holotype male (MNRJ) **37** Tergal modification of the tergite I, dorsal view, holotype male (MNRJ) **38** Tergal modification of the tergite VII, holotype male (MNRJ) **39** Subgenital plate, ventral view, holotype male (MNRJ) **40** Supra anal plate, dorsal view, holotype male (MNRJ) **41** right paraproct, dorsal view (MNRJ) **42** left phallomere, dorsal view (MNRJ) **43** median sclerite, dorsal view (MNRJ) **44** right phallomere, dorsal view (MNRJ).

General coloration dark brown, shiny (Fig. 34). Pronotum yellowish brown; central disk with scattered brown marks (Fig. 35). Head rusty yellow; frons and clypeus with occasional marks and labrum brown (Fig. 36). Antennae with basal segments pale and apical segments pigmented. Ocelli whitish. Legs with brown mark at base of coxae and margins. Abdomen dark brown with white marks from tergite I to tergite IV; tergite VII with white lateral marks; sternite orange brown.

Head. Triangular, interocular space half width of antennal insertions. Ocelli well developed. Vertex slightly exposed. Maxillary palp setose on segments 4 and 5, 3^rd^ and 5^th^ segments subequal in length; 4^th^ segment slightly smaller than both.

Thorax. Pronotum slightly convex, angulate on posterior surface, widest in medio-caudal region. Lateral flaps developed and deflexed. Legs robust with coxae wide; fore femur on anteroventral surface with row of 9 long spines, decreasing in size toward apex and ending in 3 elongate apical spines. Posteroventral surface with sparse spines, irregular, last spine apical. Fore and hind femora with spines on both margins and genicular spines. Pulvilli, arolia, and claws well developed. Tegmen well developed, reaching beyond tip of cerci. Marginal field well demarcated. Discoidal field convex and with venular arrangement. Anal field ample, convex, with six axillary veins. Wings developed; anal field fan-folded; apical triangle small.

Abdomen. Tergites I and VII modified (Figs 37 and 38). Subgenital plate asymmetrical, setose at apex and styles unequal in shape and size, inserted laterally on plate. Right style bifid and pointed, with accessory style; left style rectangular, with approximately six spines on ventral margin (Fig. 39). Supra-anal plate projected between cerci, bilobed apically, setose on margins. Cerci long (Fig. 40). Right paraproct long, L-shaped, reaching beyond half the length of the supra-anal plate, covered ventrally with sclerotized setae resembling spines (Fig. 41). Genitalia with left phallomere hook-shaped, recurved internally (Fig. 42). Median sclerite elongate, with apex slightly curved and pointed (Fig. 43); right phallomere weakly sclerotized, median portion triangular (Fig. 44).

##### Known geographical distribution.

Brazil (MT)

## Supplementary Material

XML Treatment for Xestoblatta buhrnheimi

XML Treatment for Xestoblatta rondonensis

XML Treatment for Xestoblatta mamorensis

XML Treatment for Xestoblatta roppai
